# Supplementary material for: Mutations in MDH2, Encoding a Krebs Cycle Enzyme, Cause Early-Onset Severe Encephalopathy
Source: Am J Hum Genet. 2016 Dec 15;100(1):151–9. doi: 10.1016/j.ajhg.2016.11.014 (PMC5223029; doi:10.1016/j.ajhg.2016.11.014)
Supplement: Document S1. Figures S1–S3 and Tables S1–S3 [file mmc1.pdf]

The American Journal of Human Genetics, Volume *100*

Supplemental Data

Mutations in *MDH2*, Encoding a Krebs Cycle Enzyme,

Cause Early-Onset Severe Encephalopathy

Samira Ait-El-Mkadem, Manal Dayem-Quere, Mirjana Gusic, Annabelle Chaussenot, Sylvie Bannwarth, Bérengère François, Emmanuelle C. Genin, Konstantina Fragaki, Catharina L.M. Volker-Touw, Christelle Vasnier, Valérie Serre, Koen L.I. van Gassen, Françoise Lespinasse, Susan Richter, Graeme Eisenhofer, Cécile Rouzier, Fanny Mochel, Anne De Saint-Martin, Marie-Thérèse Abi Warde, Monique G.M. de Sain-van der Velde, Judith J.M. Jans, Jeanne Amiel, Ziga Avsec, Christian Mertes, Tobias B. Haack, Tim Strom, Thomas Meitinger, Penelope E. Bonnen, Robert W. Taylor, Julien Gagneur, Peter M. van Hasselt, Agnès Rötig, Agnès Delahodde, Holger Prokisch, Sabine A. Fuchs, and Véronique Paquis-Flucklinger

SUPPLEMENTAL FIGURES

Figure S1.

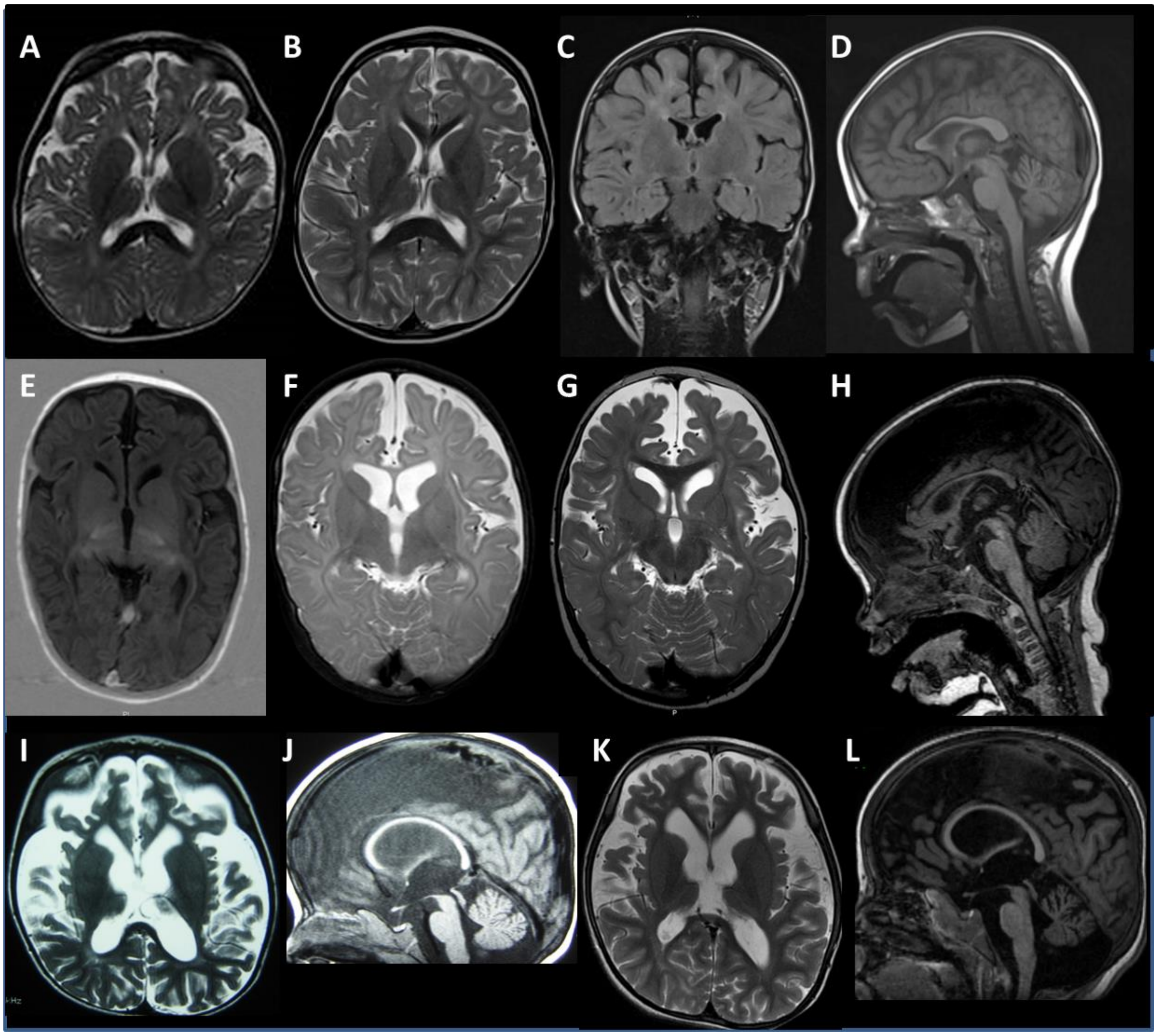

Brain MRI of Subject 1 (A-D), Subject 2 (E-H) and Subject 3 (I-L). Subject 1: Supratentorial cortical atrophy mainly in the frontal and parietal lobes at one year of age (A) and at 2.5 years of age (B-C) with thinning of the anterior part of the corpus callosum (D). Subject 2: normal MRI at 3 months of age (E). Gradual cortical and subcortical atrophy in the frontal lobes at 6 months of age (F) with parietal involvement at one year of age (G) and thinning of the genu of the corpus callosum (H). Subject 3: Cerebral cortical and subcortical atrophy at 18 months of age (I-J) associated with cerebellar atrophy at 3.5 years (K-L).

Figure S2.

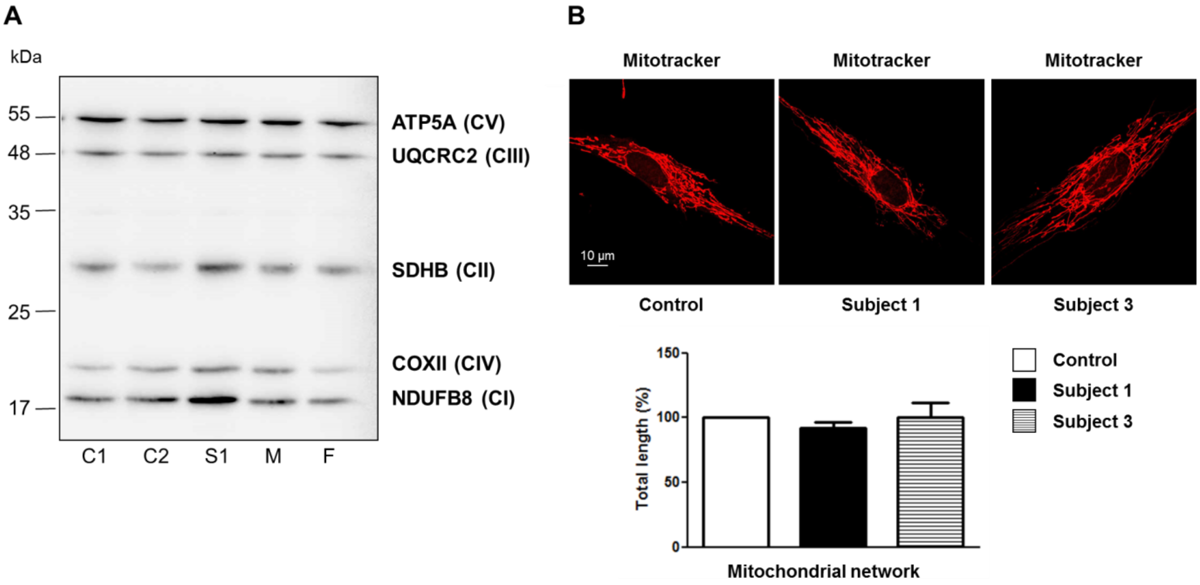

OXPHOS levels and mitochondrial network in fibroblasts from affected children. A. Representative western blot of ATP5A, UQCRC2, SDHB, COX II, and NDUFB8 performed with fibroblast lysates obtained from two control individuals (C1, C2), the affected subject (S1), his mother (M) and his father (F). A cocktail of mouse anti-human total OXPHOS complex antibodies (Abcam, ab110411) was used at 1/1000 for the blot. CI, complex I; CII, complex II ; CIII, complex III ; CVI, complex VI ; CV, complex V. **B.** Cells obtained from a control and the affected subjects (S1 and S3) were analyzed by confocal microscopy using Mitotracker Red (upper panel) as previously described.[1] Mitochondrial phenotypes were quantified for 35 randomly-selected individual cells per each studied fibroblast cell line from 3 independent experiments (lower panel). The data obtained were used to calculate the total length of the mitochondrial network per cell. Differences between the cell lines were analyzed by Student's t-test: ns, non significant.

Figure S3.

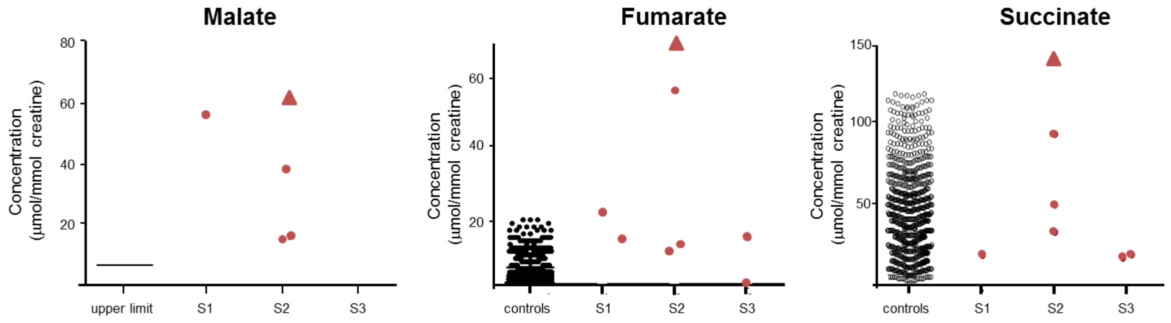

Urinary malate, fumarate and succinate excretion in the affected subjects. S1-3, Subjects 1-3; reference values (malate: from literature, fumarate n=728 and succinate n>1000) representing the 95% confidence interval of all analyses performed in Utrecht metabolic laboratory in boys (age 0-4 years) from January 2010 to May 2016. Circle, in normal conditions; triangle, during metabolic decompensation.

SUPPLEMENTAL TABLES

Table S1. Respiratory chain analysis in samples from Subjects 1 and 3. Spectrophotometric analysis on muscle (A), liver (B) of Subject 1 and muscle of Subject 3 (C). Spectrophotometric analysis on fibroblasts of Subjects 1 and 3 cultivated in glucose (D) and galactose (F) medium. Polarographic analysis of the respiratory chain enzyme activities in fibroblasts of Subjects 1 and 3 cultivated in glucose (E) and in galactose medium (G). Results are expressed as extreme absolute values or absolute values for controls or affected subjects, respectively. Lower values are highlighted in grey. nd, not done. G3P = glycerol 3-phosphate. CS = citrate synthase. LDH = lactate dehydrogenase. Spectrophotometric measurements and polarographic studies were performed as previously described.[2]

A

SPECTROPHOTOMETRIC ANALYSIS ON MUSCLE							
Enzymatic activities	I	II	III	IV	V	CS	LDH
Control values (nmol/min/mg of proteins)	10-32	20-65	94-236	89-347	32-89	82-234	1259-7234
Subject 1	22.5	36.9	232.9	255.7	50.3	175.6	4106

B

SPECTROPHOTOMETRIC ANALYSIS ON LIVER							
Enzymatic activities	I	II	III	IV	V	CS	LDH
Control values (nmol/min/mg of proteins)	13-41	78-267	124-246	146-316	41-117	43-107	736-2359
Subject 1	35.5	157.0	154.0	246.5	39.7	77.8	1438

C

SPECTROPHOTOMETRIC ANALYSIS ON MUSCLE							
Enzymatic activities	I	II	III	IV	V	CS	LDH
Control values (nmol/min/mg of proteins)	40-95	66-153	925-2068	418-1210	165-414	82-234	1259-7234
Subject 3	68.0	nd	1820.0	920.0	122.0	nd	nd

D

SPECTROPHOTOMETRIC ANALYSIS ON FIBROBLASTS GROWN IN GLUCOSE MEDIUM							
Enzymatic activities	I	II	III	IV	V	CS	LDH
Control values (nmol/min/mg of proteins)	9.0-27.1	18.5-54.0	57.4-176.2	109.9-350.0	22.0-46.2	74.7-161.1	2642-6971
Subject 1	13.3	26.8	100.3	229.9	45.0	137.3	4022
Control values (nmol/min/mg of proteins)	27-44	56-83	547-846	285-436	53-84	74.7-161.1	2642-6971
Subject 3	27.0	145.0	1140.0	678.0	95.0	188.6	4601

E

POLAROGRAPHIC ANALYSIS ON FIBROBLASTS GROWN IN GLUCOSE MEDIUM				
Oxygen consumption	Intact cells	Digitonin permeabilized cells		
		Glutamate+Malate	Succinate	G3P
Control values (nmol O2/min/mg of proteins)	5.90-13.80	8.00-16.60	8.00-15.80	4.90-13.50
Subject 1	10.40	8.82	8.66	9.70
Subject 3	9.85	12.52	10.75	9.95

F

SPECTROPHOTOMETRIC ANALYSIS ON FIBROBLASTS GROWN IN GALACTOSE MEDIUM							
Enzymatic activities	I	II	III	IV	V	CS	LDH
Control values (nmol/min/mg of proteins)	15.2-20.1	28.2-48.0	88.8-143.0	181.7-315.4	22.7-47.5	124.8-225.0	3100-6100
Subject 1	18.3	29.8	114.1	253.4	32.5	134.4	5816
Subject 3	23.4	62.6	224.8	362.8	48.6	238.0	4019

G

POLAROGRAPHIC ANALYSIS ON FIBROBLASTS GROWN IN GALACTOSE MEDIUM				
Oxygen consumption	Intact cells	Digitonin permeabilized cells		
		Glutamate+Malate	Succinate	G3P
Control values (nmol O2/min/mg of proteins)	5.58-17.44	8.16-13.45	8.60-10.97	5.22-12.91
Subject 1	15.51	11.00	10.03	9.49
Subject 3	29.27	18.83	16.87	17.65

Table S1

Table S2. Bioinformatics analysis of *MDH2* missense variants.

* : UCSC GRCh37/hg19 assembly

D: damaging or disease causing

CADD: Combined Annotation Dependent Depletion

GERP: Genomic Evolutionary Rate Profiling

PhyloP: phylogenetic p-values

# Chr	Genomic position*	NM-ID	cDNA change	Protein change	SIFT score	SIFT prediction	Polyphen2 _hdiv score	Polyphen2 _hdiv prediction	MutationTaster score	MutationTaster prediction	CAAD_Phred	GERP_RS	PhyloP score
7	75684190	NM_005918	G109A	G37R	0,99	D	1	D	1	D	34	5,83	2,76
7	75687365	NM_005918	C398T	P133L	1	D	1	D	1	D	18,3	5,56	2,9
7	75692897	NM_005918	C620T	P207L	1	D	1	D	1	D	21,8	5,69	2,69

Table S3. Sequences of primers used for Sanger sequencing and complementation studies

Name	Sequence
Primers used for Sanger sequencing of MDH2 variants	
MDH2-Ex2F	gaggcccagtgcaacattat
MDH2-Ex2R	gaaacccccgtctctaaaca
MDH2-Ex4F	atttcctgtaacagctggcg
MDH2-Ex4R	gcacatcagcagcctttg
MDH2-Ex6F	ggcagctgtgacatgttttac
MDH2-Ex6R	Aggccttctccagtctacc
Primers used for molecular cloning of human MDH2 cDNA	
MDH2-BclI-F	ttccatgatcacccgctccagccatgct
MDH2-NotI-R	actttgcggccgctcacttcagggtctt
Primers used for Sanger sequencing of human MDH2 cDNA	
MDH2-cDNA-F1	gacgacctgttcaacaccaa
MDH2-cDNA-R1	gtgttggctctgacgatgtc
MDH2-cDNA-F2	tggatccagctcgagtcaa
MDH2-cDNA-R2	gcggtgtggagaagtaggta
MDH2-cDNA-F3	aagggcatcgagaagaacct
MDH2-cDNA-R3	aggcggctcaccaaggggc
Primers used for yeast studies	
F1-MDH1	ctttaaaggacacagacatggttttaattccggatccccgggttaattaa
R1-MDH1	tttcgatattcttcttcaaggtttctttacgaattcgagctcgtttaaac
MDH2-Eco	cggatttccgatgctctccgccctcgtcc
MDH2-Hind	cccaagcttgggtcacttcagggtcttcacg
MDH1-Recfw	ctataactacaaaaaacacatacaggaattagcttacgcgtcgagcccggaattccgatgttgtcaagagtagctaaac
MDH1-RecRev	aaggaaaaacgttcattgttccttattcagttagctagctcccaagcttgggtcacttcctatttactagcaacaaagttg
mdh1-P128Lfw	accgccgaatccgctctcaatgctgccattctg
mdh1-P128LRev	cagaatggcagcattgagagcggattcggcggt
mdh1-P202Lfw	ggtattaccatcatcctattgatttcgcaaaca
mdh1-P202LRev	tgtttgcgaaatcaataggatgatggtaatacc
mdh1-G30Rfw	gcaggcggtggtattggacaaccattg
mdh1-PG30RRev	caatggttgtccaataccaccgcctgc

SUPPLEMENTAL REFERENCES

1. Bannwarth, S., Ait-El-Mkadem, S., Chaussenot, A., Genin, E.C., Lacas-Gervais, S., Fragaki, K., Berg-Alonso, L., Kageyama, Y., Serre, V., Moore, D.G., et al. (2014). A mitochondrial origin for frontotemporal dementia and amyotrophic lateral sclerosis through CHCHD10 involvement. Brain *137*, 2329–2345.

2. Rustin, P., Chretien, D., Bourgeron, T., Gérard, B., Rötig, A., Saudubray, J.M., and Munnich, A. (1994). Biochemical and molecular investigations in respiratory chain deficiencies. Clin. Chim. Acta Int. J. Clin. Chem. *228*, 35–51.
