# Supplementary material for: Mutations in MDH2, Encoding a Krebs Cycle Enzyme, Cause Early-Onset Severe Encephalopathy
Source: Am J Hum Genet. 2016 Dec 15;100(1):151–9. doi: 10.1016/j.ajhg.2016.11.014 (PMC5223029; doi:10.1016/j.ajhg.2016.11.014)
Supplement: Document S2. Article plus Supplemental Data [file mmc2.pdf]

Mutations in *MDH2*, Encoding a Krebs Cycle Enzyme, Cause Early-Onset Severe Encephalopathy

Samira Ait-El-Mkadem,[1,2,18] Manal Dayem-Quere,[1,2,18] Mirjana Gusic,[3,4,18] Annabelle Chaussenot,[1,2] Sylvie Bannwarth,[1,2] Bérengère François,[2] Emmanuelle C. Genin,[2] Konstantina Fragaki,[1,2] Catharina L.M. Volker-Touw,[5] Christelle Vasnier,[6] Valérie Serre,[7] Koen L.I. van Gassen,[5] Françoise Lespinasse,[2] Susan Richter,[8] Graeme Eisenhofer,[8] Cécile Rouzier,[1,2] Fanny Mochel,[9] Anne De Saint-Martin,[10] Marie-Thérèse Abi Warde,[10] Monique G.M. de Sain-van der Velde,[11] Judith J.M. Jans,[11] Jeanne Amiel,[12] Ziga Avsec,[13] Christian Mertes,[13] Tobias B. Haack,[3,4] Tim Strom,[3,4] Thomas Meitinger,[3,4] Penelope E. Bonnen,[14] Robert W. Taylor,[15] Julien Gagneur,[13] Peter M. van Hasselt,[16] Agnès Rötig,[17] Agnès Delahodde,[6] Holger Prokisch,[3,4] Sabine A. Fuchs,[11] and Véronique Paquis-Flucklinger[1,2,*]

MDH2 encodes mitochondrial malate dehydrogenase (MDH), which is essential for the conversion of malate to oxaloacetate as part of the proper functioning of the Krebs cycle. We report bi-allelic pathogenic mutations in *MDH2* in three unrelated subjects presenting with early-onset generalized hypotonia, psychomotor delay, refractory epilepsy, and elevated lactate in the blood and cerebrospinal fluid. Functional studies in fibroblasts from affected subjects showed both an apparently complete loss of MDH2 levels and MDH2 enzymatic activity close to null. Metabolomics analyses demonstrated a significant concomitant accumulation of the MDH substrate, malate, and fumarate, its immediate precursor in the Krebs cycle, in affected subjects' fibroblasts. Lentiviral complementation with wild-type *MDH2* cDNA restored MDH2 levels and mitochondrial MDH activity. Additionally, introduction of the three missense mutations from the affected subjects into *Saccharomyces cerevisiae* provided functional evidence to support their pathogenicity. Disruption of the Krebs cycle is a hallmark of cancer, and *MDH2* has been recently identified as a novel pheochromocytoma and paraganglioma susceptibility gene. We show that loss-of-function mutations in *MDH2* are also associated with severe neurological clinical presentations in children.

Mitochondrial diseases, caused by respiratory chain (RC) deficiency, encompass a wide range of clinical manifestations. They mainly affect organs with high-energy requirements, such as the brain. They are also increasingly recognized as causes of refractory epilepsy, which is consistently associated with progressive neurologic deterioration.[1] Genetic diagnosis of RC disorders remains challenging because of the involvement of mitochondrial DNA (mtDNA) or nuclear DNA. In addition, RC dysfunction might be the primary cause of symptoms or secondary to other disorders. The Krebs cycle is intimately linked to the RC, and Krebs cycle defects are among the diseases that mimic or cause RC deficiencies. However, human diseases associated with defects in the Krebs cycle are very rare, putatively because of the cycle's essential function in cellular energy metabolism.

Here, we show that mutations in *MDH2* (MIM: 154100), encoding the Krebs cycle enzyme mitochondrial malate dehydrogenase (MDH), are responsible for severe neurological manifestations in children. We report bi-allelic *MDH2* variants in three unrelated subjects presenting with an early-onset mitochondrial phenotype comprising generalized hypotonia, psychomotor delay, and refractory epilepsy. All affected individuals were independently identified by whole-exome sequencing (WES). Two of them were "matched" by GeneMatcher, a web-based tool for connecting researchers and clinicians with shared interests in identical genes.[2] The third subject was identified

[1]Department of Medical Genetics, National Centre for Mitochondrial Diseases, Nice Teaching Hospital, 06202 Nice, France; [2]Nice Sophia-Antipolis University, CNRS UMR 7284, INSERM U1081, Institute for Research on Cancer and Aging, Nice, 06107 Nice, France; [3]Institute of Human Genetics, Technical University of Munich, 81675 München, Germany; [4]Institute of Human Genetics, Helmholtz Zentrum München, 85764 Neuherberg, Germany; [5]Department of Genetics, University Medical Center Utrecht, 3584 Utrecht, the Netherlands; [6]Institute for Integrative Biology of the Cell (I2BC), Commissariat à l'Énergie Atomique et aux Énergies Alternatives, CNRS, Université Paris-Sud, Université Paris-Saclay, 91198 Gif-sur-Yvette Cedex, France; [7]CNRS UMR 7592, Jacques Monod Institute, Paris Diderot University, 75205 Paris, France; [8]Institute of Clinical Chemistry and Laboratory Medicine, University Hospital Carl Gustav Carus, Medical Faculty Carl Gustav Carus, Technische Universität Dresden, Fetscherstrasse 74, 01307 Dresden, Germany; [9]INSERM U1127, CNRS UMR 7225, Sorbonne Universités, l'Université Pierre et Marie Curie (Paris 06) UMR S1127, Institut du Cerveau et de la Moelle Épinière Department of Genetics, Pitié-Salpêtrière University Hospital, 75013 Paris, France; [10]Service de Neurologie Pédiatrique, Centre de Référence pour les Épilepsies Rares, Centre Hospitalier Universitaire de Strasbourg, 67098 Strasbourg, France; [11]Laboratory of Metabolic Diseases, University Medical Center Utrecht, 3584 Utrecht, the Netherlands; [12]Department of Genetics, Hôpital Necker-Enfants Malades, 75015 Paris, France; [13]Department of Informatics, Technical University of Munich, 85748 Garching, Germany; [14]Department of Molecular and Human Genetics, Baylor College of Medicine, Houston, TX 77030, USA; [15]Wellcome Trust Centre for Mitochondrial Research, Institute of Neuroscience, Newcastle University, NE2 4HH Newcastle upon Tyne, UK; [16]Department of Metabolic Diseases, University Medical Center Utrecht, 3584 Utrecht, the Netherlands; [17]INSERM U1163, Université Paris Descartes-Sorbonne Paris Cité, Institut Imagine, 75015 Paris, France
[18]These authors contributed equally to this work
*Correspondence: paquis@hermes.unice.fr
http://dx.doi.org/10.1016/j.ajhg.2016.11.014.
© 2017 The Author(s). This is an open access article under the CC BY license (http://creativecommons.org/licenses/by/4.0/).

Table 1. Genetic and Clinical Findings in Subjects with Bi-allelic *MDH2* Variants

	Subject 1	Subject 2	Subject 3
Gender	male	male	male
Family history	no	no	no
Consanguinity	no	no	no
Age at last clinical examination	4.5 years	died at 1.5 years	7.5 years (lost view)
MDH2 mutations	c.398C>T (p.Pro133Leu)	c.398C>T (p.Pro133Leu)	c.109G>A (p.Gly37Arg)
	c.620C>T (p.Pro207Leu)	c.596delG (p.Gly199Alafs*10)	c.398C>T (p.Pro133Leu)
Age at onset	5 months	neonatal	neonatal
Initial symptom	hypotonia, no head control	seizures	hypotonia
Refractory epilepsy	+ (partial, afterward myoclonic; onset at 7 months)	+ (generalized tonic and spasms; onset at 2 months)	+ (myoclonic epilepsy and generalized tonic; onset ?)
Hypotonia	+ (marked, mainly axial and in the lower limbs)	+ (axial)	+
Developmental delay	+	+	+
Head control	10 months	not acquired at 6 months	12 months
Sitting position	18 months	not acquired at 12 months	−
Crawling	18 months	not acquired at 12 months	no
Good eye contact	yes	yes	no
Language	not acquired	babbling at 12 months	not acquired
Muscle weakness	+	+	+
Failure to thrive	+	−	+
Age at onset	7 months	−	?
Gastrostomy (age)	+ (3 years)	−	+ (?)
Last examination	4 years	18 months	7.5 years
Length	< −2 SDs	+ 1 SD	−2 SDs
Head circumference	< −2 SDs	+ 1 SD	+2 SDs
Weight	< −3 SDs	+ 1 SD	−2.5 SDs
Movement disorders	dystonia and dyskinesia	−	dystonia
Obstinate constipation	+	+	−
Ophthalmologic examination (age)	retinitis pigmentosa (4 years), strabismus (5 months)	strabismus (1 year)	?
Pyramidal signs	+	+	−
Deep tendon reflexes	decreased	N	−
Plantar responses	bilateral extensor	bilateral extensor after 1 year	normal
Other findings	−	−	two supernumerary nipples, von Willebrand disease, CCAM
Ketogenic diet (onset)	+ (3 years)	+ (18 months)	+ (3 years)
Response to Ketogenic diet	reduction epileptic seizure frequency	reduction epileptic seizure frequency	?
Evolution	alive at 5 years	died at 1.5 years (secondary to metabolic decompensation)	alive at 12 years
Brain MRI abnormalities	+ (atrophy of the anterior part of the CC, delayed myelination of the frontal white matter, frontal and parietal atrophy, elevated lactic acid peak on MRS)	+ (delayed myelination of the genu of the CC, cortical and subcortical atrophy of the frontal lobes)	+ (cerebral cortical and subcortical atrophy, cerebellar atrophy, elevated lactic acid peak on MRS)

(*Continued on next page*)

Table 1. *Continued*

	Subject 1	Subject 2	Subject 3
**Lactate Concentration**			
Plasma (N < 2.20 mmol/l)	elevated (3.0)	elevated (5.7)	elevated (2.8)
L/P ratio (N < 18)	elevated (63)	elevated (23)	elevated (20)
CSF (N < 2.10 mmol/l)	elevated (2.48)	elevated (3.3)	ND
**Krebs Cycle Intermediates**			
Malate (N < 7 μmol/mmol creatine)	elevated (56)	elevated (15–38)	ND
Fumarate (N < 14 μmol/mmol creatine)	elevated (20)	N or elevated (9–55)	N
Succinate	N	N	N
**RC Activity**			
Muscle	N	ND	N
Liver	reduced CV activity	ND	ND
Fibroblasts	N	ND	reduced CI activity

Abbreviations are as follows: +, present; −, absent; ?, unknown; N, normal; ND, not done; SD, standard deviation; CCAM, congenital cystic adenomatoid malformation; MRS, magnetic resonance spectroscopy; CC, corpus callosum; L/P, lactate/pyruvate; CSF, cerebrospinal fluid; RC, respiratory chain; CV, complex V; and CI, complex I.

independently and was matched within GENOMIT, an European network of researchers with an interest in mitochondrial genetic disorders.

Informed consent for diagnostic and research studies was obtained for all subjects in accordance with the Declaration of Helsinki protocols and was approved by local ethics committees. Subject 1 (S1) in family F (F1:II.2) is the second, male child of healthy, non-consanguineous French parents. Pregnancy and birth were both unremarkable. At 5 months of age, he presented with marked hypotonia and absence of head control (detailed in Table 1). His overall disease course was characterized by psychomotor delay with partial epileptic seizures that rapidly evolved toward refractory myoclonic epilepsy, failure to thrive, and obstinate constipation. At 3 years of age, growth remained problematic despite tube feeding through percutaneous gastrostomy, and he presented with generalized muscle weakness (predominant in the lower limbs) with marked muscle atrophy, severe hypotonia, and abnormal movements with dyskinesia. At 4 years, retinitis pigmentosa was noted. Brain magnetic resonance imaging (MRI) showed nonspecific findings including atrophy of the anterior part of the corpus callosum, delayed myelination of the frontal white matter, and cortical, frontal, and parietal atrophy (Figure S1). The clinical phenotype, combined with elevated lactate concentrations in both plasma and cerebrospinal fluid (CSF), was evocative of a mitochondrial disease. A slight decrease in complex V activity was found in the liver, whereas muscle tissue showed no signs of mitochondrial dysfunction (Tables S1A and S1B). We observed no evidence of mtDNA rearrangements in either muscle or liver tissue and excluded mtDNA point mutations by using targeted next-generation sequencing protocols. WES via previously described methodologies and bioinformatic filtering pipelines[3] identified compound-heterozygous missense variants in *MDH2* (GenBank: NM_005918.3): c.398C>T (p.Pro133Leu) and c.620C>T (p.Pro207Leu). Familial segregation studies showed that the c.398C>T variant was inherited from the father (F1:I.1) and the c.620C>T variant was inherited from the mother (F1:I.2), whereas a healthy sister (F1:II.1) was a heterozygous carrier of the paternal (c.398C>T) variant only (Figure 1A).

Subject 2 (S2) (F2:II.1) was the first male child of healthy, unrelated Dutch parents (F2:I.1 and I.2). He was born after an uncomplicated pregnancy and presented during the neonatal period with obstinate constipation and positional preference of his head (Table 1). At 2 months, he developed refractory epilepsy with generalized tonic seizures and epileptic spasms but had normal brain MRI (Figure S1). At 6 months, examination showed axial hypotonia, head lag, and developmental delay in the absence of failure to thrive. Repeat brain MRI showed delayed myelination of the genu of the corpus callosum and cortical and subcortical atrophy of the frontal lobes (Figure S1). Examination at 1 year of age demonstrated muscle weakness, and he was not able to sit. Ophthalmologic examination was normal. Metabolic screening showed elevated lactate concentrations in both blood and CSF (Table 1). In a clinical examination at 20 months of age, he presented with coughing, vomiting, tachypnea, and impaired consciousness associated with severe metabolic acidosis (blood pH = 6.97 [normal 7.4] and plasma lactate concentration = 9.0 mmol/l [normal range < 2.20mmol/l]). The urinary organic acid profile was highly abnormal with strongly increased excretion of ketone bodies, lactate, pyruvate,

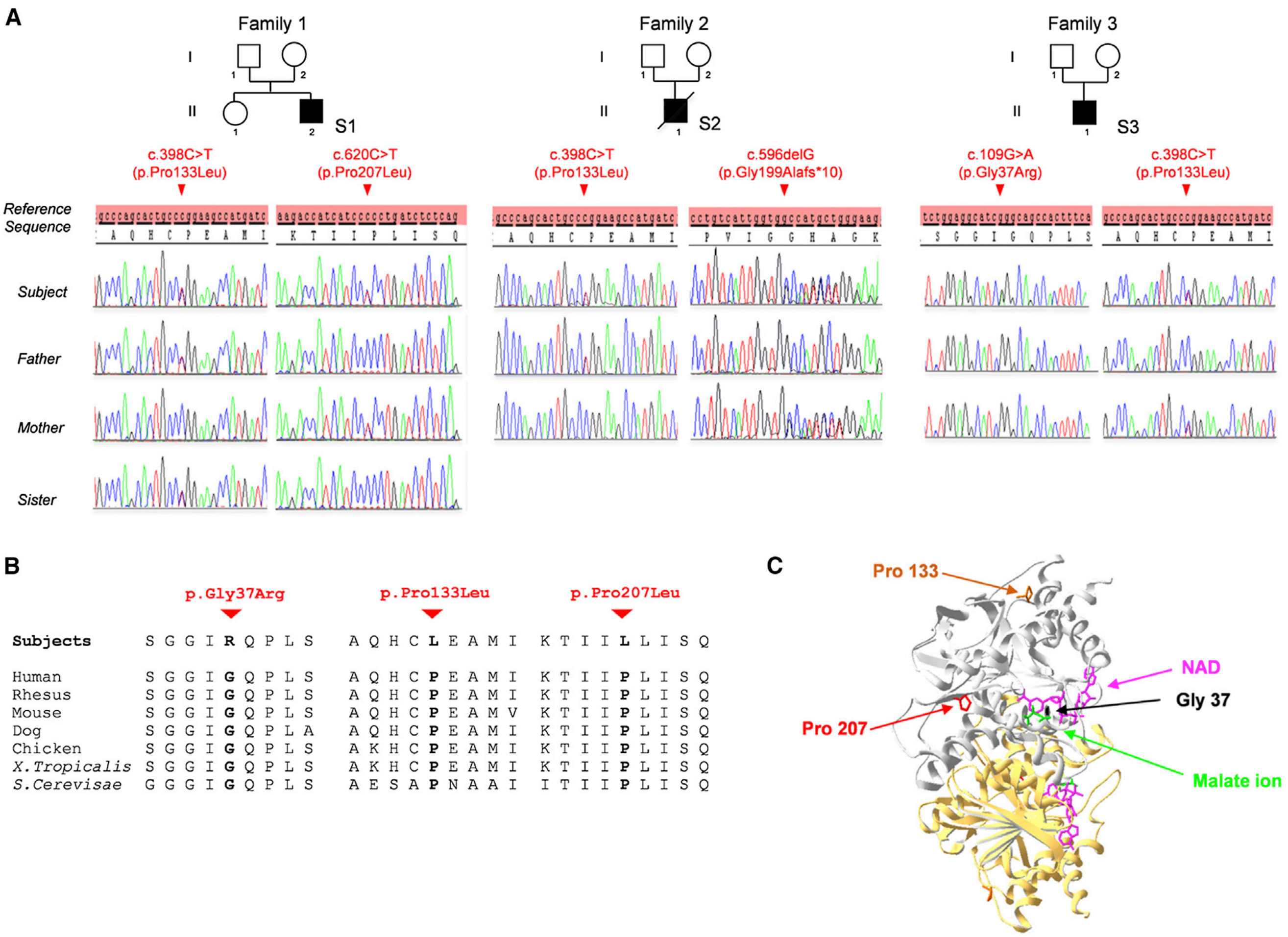

Figure 1. *MDH2* Mutations in Three Unrelated Affected Subjects
(A) Pedigrees and sequence chromatograms showing variant phenotypes and segregation through the subjects' families.
(B) Cross-species conservation of the MDH2 sequence flanking the altered Gly37, Pro133, and Pro207 amino acids.
(C) Three-dimensional representation of the crystal structure of human MDH2 (PDB: 2DFD, residues 24–337), shown as a homodimer with one molecule in gray and a second in yellow. Green and fuchsia sticks illustrate malate ions and NAD, respectively. The mutated residues are highlighted in black (Gly37), orange (Pro133), and red (Pro207).

malate, fumarate, and 3-methylglutaconic acid. During the course of his hospital admission, he developed severe pulmonary hypertension requiring resuscitation, although a thorough cardiac evaluation revealed no underlying heart defect. He died 1 week later upon withdrawal of ventilatory support. Molecular screening, including mtDNA sequencing, *POLG* sequencing, and epilepsy gene-panel analysis failed to establish a molecular diagnosis. WES identified two heterozygous *MDH2* variants: a paternally inherited c.398C>T (p.Pro133Leu) missense variant, which was also found in S1, and a maternally inherited c.596delG (p.Gly199Alafs*10) nonsense variant (Figure 1A).

Independently, *MDH2* was prioritized by GenePROF, a machine-learning algorithm we have recently developed to prioritize potential disease-causing mutations according to variant frequency and functional gene annotation obtained from molecular-pathway databases (manuscript in preparation). GenePROF ranked *MDH2* as the most likely disease-causing gene for an unrelated affected subject (S3, F3:II.1) who also carries a maternally inherited c.398C>T (p.Pro133Leu) *MDH2* variant (Figure 1A). This individual is also heterozygous for a de novo c.109G>A (p.Gly37Arg) variant that is not present in either parent (F3:I.1 or I.2). Paternity was confirmed by genotype analysis of seven informative microsatellites. This child was born at term to healthy, unrelated French parents and presented with severe hypotonia, macrocephaly, macrosomia, and two supernumerary nipples (Table 1). His overall disease course was characterized by psychomotor delay with refractory epilepsy, dystonia, and failure to thrive despite tube feeding through percutaneous gastrostomy. Lactate was elevated in blood (2.8 mmol/L [normal range < 2.20mmol/L]), consistent with a lactate peak on magnetic resonance spectroscopy (MRS) (data not shown). Marked cerebral and cerebellar atrophy were observed on brain MRI at the age of 4 years (Figure S1). A slight decrease in complex V activity was found in muscle tissue (Table S1C).

Multiple lines of evidence provided by bioinformatics analyses support the pathogenicity of the missense

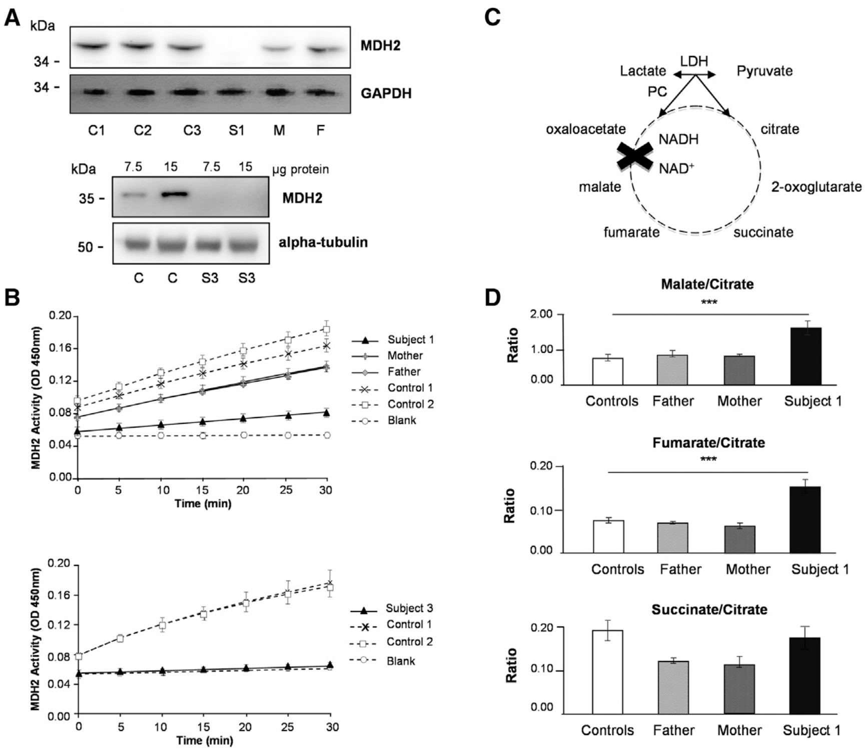

Figure 2. ***MDH2*** **Mutations Cause Loss of MDH2 Levels and Enzymatic Activity**
(A) Western blot analysis with anti-MDH2 antibodies in fibroblasts from subject 1 (S1, top) and subject 3 (S3, bottom). Additional abbreviations are as follows: C1–C3, control individuals; M, mother of S1; and F, father of S1. Commercial MDH2-specific antisera at 1:250 (HPA019716, Sigma-Aldrich) or 1:1,000 (8610S, Cell Signaling Technology) were used for the upper and lower panels, respectively. GAPDH and α-tubulin were used as loading controls.
(B) Representation of MDH2 activity, measured with the Mitochondrial Malate Dehydrogenase (MDH2) Activity Assay Kit (Ab119693, Abcam), in the fibroblasts from S1, his parents, and control individuals (top) and in those from S3 and control individuals (bottom). Data represent three independent experiments performed in duplicate.
(C) Schematic of the Krebs cycle.
(D) Metabolite ratios assessed by liquid chromatographic tandem-mass spectrometry in fibroblasts from S1 are compared with those from his parents and control individuals. Experiments were performed as previously described.[4] Differences between fibroblast cells were analyzed by Student's t test: ***$p < 0.001$ (extremely significant).

variants identified in these subjects. The c.620C>T variant detected in S1 was absent from public databases, whereas the c.109G>A variant detected in S3 was found two times, and the c.398C>T variant, present in all subjects, was listed seven times only in a heterozygous state in ~120,000 alleles from the Exome Aggregation Consortium (ExAC) Browser. Additionally, the three different missense variants found in affected subjects alter highly conserved amino acid residues (Figure 1B), and bioinformatics analyses predicted these variants as pathogenic (Table S2). The dimeric protein was co-crystallized with malate ion and nicotinamide-adenine-dinucleotide (NAD) for visualization of the structure and analysis of the structural consequences of MDH2 variants (Figure 1C). Gly37 is located in the NAD binding pocket; the mutation from this residue to arginine is likely to alter the binding of the coenzyme as a result of steric hindrance. Pro133 is located in a turn between an α helix and a β strand. Pro207 is located in a β strand. The turns are almost exclusively found on the surface of proteins and often contain proline residues that form a rigid backbone, as predicted for Pro133. On the contrary, proline tends to be excluded from β strands, but it can be situated at the ends of this motif, like for Pro207. In this case, proline imposes its own kind of secondary structure with a confined ϕ angle, creating a twist in the β strand. Thus, Pro133 and Pro207 are predicted to have structural roles by contributing to the overall architecture of MDH2. Consequently, the p.Pro133Leu and p.Pro207Leu variants might result in destabilization of protein regions necessary for functional protein folding.

We used primary fibroblast cultures established from S1 and S3 to confirm the role of *MDH2* variants in disease. Western blotting showed that MDH2 levels were not detectable in either S1 or S3 fibroblasts, supporting the notion that corresponding *MDH2* variants adversely affect the stability of the protein (Figure 2A). In addition, MDH enzymatic activity was markedly decreased in that it showed severe MDH deficiency (Figure 2B). The activity was notably lower in cells obtained from the parents of S1 (F1: I.1 and I.2, heterozygous for the c.398C>T and c.620C>T variants, respectively) than in control fibroblasts. This result suggests that each mutation individually has a deleterious effect. As expected from MDH function in the Krebs cycle (Figure 2C), ratios of malate and its direct precursor, fumarate, to citrate were significantly higher in S1 cells than in control fibroblasts without accumulation of the earlier Krebs cycle precursor, succinate (Figure 2D). MDH activity appeared sufficient in cells from the parents of S1 to maintain normal malate/citrate and fumarate/citrate ratios.

Next, we expressed wild-type human *MDH2* cDNA in *MDH2*-deficient S1 fibroblasts by using a lentiviral-based transduction system, leading to the restoration of both MDH2 levels and enzyme activity (Figures 3A and 3B). To definitively substantiate that the disease-segregating missense *MDH2* variants indeed cause MDH2 deficiency, we used the yeast *Saccharomyces cerevisiae* as a proven system for modeling mitochondrial-disease-causing mutations.[5] In yeast, three genes encode MDH enzymes. *MDH1* codes for the mitochondrial MDH enzyme, *MDH2*

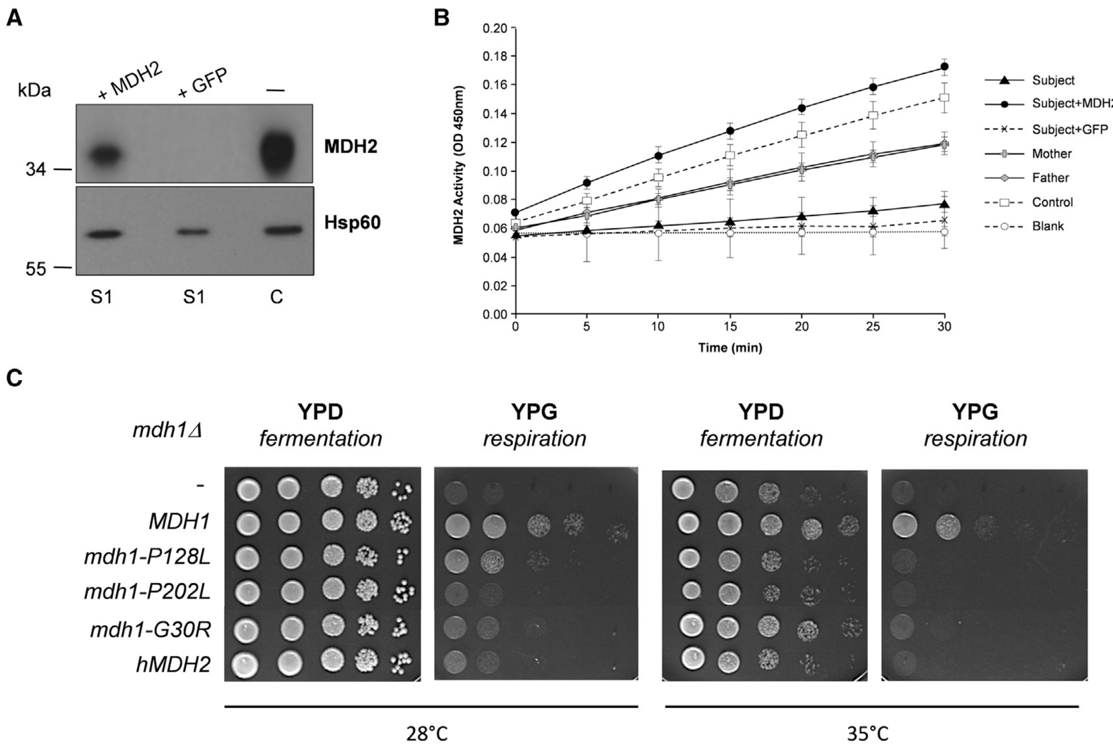

Figure 3. Functional Complementation Showing the Pathogenicity of MDH2 Variants
(A and B) Functional complementation of fibroblasts. The human full-length *MDH2* cDNA was cloned into the pHR-SIN-CSGW dlNotI vector by PCR (Table S3). Lentiviral particles were produced in HEK293T cells. Then, fibroblasts were infected with viral supernatant expressing either *MDH2* cDNA or eGFP (as a control). (A) Restoration of MDH2 levels in S1 fibroblasts transduced with wild-type *MDH2* cDNA as determined by western blot with anti-MDH2 antibody. No restoration was observed when cells were transduced with GFP cDNA (C, non-transduced control fibroblasts). (B) Restoration of MDH2 activity in S1 fibroblasts transduced with wild-type *MDH2* cDNA (two independent experiments performed in duplicate). No restoration was observed when cells were transduced with GFP cDNA. The MDH2 activity of non-transduced fibroblasts from parents and a control individual is also shown.
(C) Functional complementation assay of the yeast *mdh1Δ*-null mutant. Human *MDH2* cDNA was amplified with the corresponding primers (Table S3) and cloned into the centromeric expression plasmid pYX122 (constitutive promoter *TPI*, Addgene). The yeast wild-type *MDH1* and the *mdh1* mutated sequences corresponding to the three missense mutations were obtained by PCR (Table S3), and the corresponding fragments were introduced into the linearized pYX122 vector by co-transformation of the *mdh1Δ* strain (BY4741, Open Biosystems) and homologous recombination. Plasmids from the transformants were extracted and sequenced. These plasmids were then reintroduced into the *mdh1Δ* strain for testing their complementation capability. Growth of *mdh1Δ* (−) transformed with wild-type *MDH1*, mutated *mdh1-P128L*, *mdh1-P202L*, or *mdh1-G30R*, or wild-type *hMDH2* plasmids was tested either on glucose (YPD, fermentable carbon source) or on glycerol (YPG, non-fermentable carbon source). Drop dilution growth tests were performed at 1/10 dilution steps and incubated on YPD or YPG plates for 2 days at 28°C or 35°C.

codes for the cytoplasmic enzyme, and *MDH3* encodes the peroxisomal form. The human Gly37, Pro133, and Pro207 residues (corresponding to Gly30, Pro128, and Pro202, respectively, in yeast MDH1) are all conserved between the two species (Figure 1B). The expression of wild-type human *MDH2* cDNA failed to complement the absence of growth of the *mdh1Δ* yeast strain on non-fermentable medium despite a high degree of conservation (54% identity) between yeast and human proteins (Figure 3C). We evaluated whether the lack of complementation of the *mdh1Δ* strain by human MDH2 was due to a failure to import MDH2 into yeast mitochondria. To this end, we changed the MDH2 mitochondrial targeting sequence (MTS) by the first 69 amino acids of subunit 9 of the F_0 ATPase of *Neurospora crassa* (Pre Su9), a well-characterized sequence that targets the mitochondrial matrix in yeast.[6] Expression of the fusion MDH2 (Pre Su9-MDH2) allowed growth of the *mdh1Δ* cells on non-fermentable medium, indicating that the human MDH2 MTS is not recognized by yeast as a functional MTS and that once inside mitochondria, MDH2 can substitute for MDH1 (data not shown). Consequently, we introduced the amino acid changes into the yeast sequence. The *MDH1*, *mdh1*^{G30R}, *mdh1*^{P128L}, and *mdh1*^{P202L} constructs were transformed into the *mdh1Δ*-null mutant (Figure 3C). As expected, expression of wild-type yeast *MDH1* complemented the *mdh1Δ* strain. A clear growth defect was observed for all *mdh1Δ*/*mdh1*^{mut} strains, thus confirming the deleterious nature of the human *MDH2* variants.

Given that the Krebs cycle is intimately linked to the RC, we investigated mitochondrial RC enzyme activities in *MDH2*-deficient fibroblasts. Spectrophotometric analysis revealed no obvious abnormalities in S1 cells grown in glucose medium, but complex I activity was lower than in one of the other complexes in fibroblasts from S3 (Table S1D). Polarographic analysis showed intact cell respiration

and mitochondrial substrate oxidation in digitonin (0.004%)-permeabilized cells from both subjects (Table S1E). Identical experiments were performed on fibroblasts grown in a glucose-free medium containing galactose as previously described;[7] galactose is a carbon source that feeds the glycolytic pathway with low efficiency, and as such, cells are forced to rely predominantly on OXPHOS for ATP production. Cells with severe RC defects fail to survive under these conditions, whereas fibroblasts with a milder deficiency are able to grow.[8] No significant differences were observed between S1, S3, and control cells assessed by both spectrophotometry and polarography (Tables S1F and S1G). We also observed no decrease in OXPHOS levels in S1 cells, as judged by semiquantitative western blotting (Figure S2A). Assessment of the mitochondrial network (by confocal microscopy using MitoTracker staining) in cells from S1 and S3 showed a typical filamentous interconnected network similar to that in control fibroblasts both in glucose (Figure S2B) and in galactose medium (data not shown).

MDH2 encodes the enzyme MDH, which catalyzes the reversible oxidation of malate to oxaloacetate by utilizing the NAD-NADH cofactor system in the Krebs cycle. In addition, MDH2 plays an essential role in the malate-aspartate NADH shuttle, which is the main pathway whereby reducing equivalents from cytosolic NADH are transferred to mitochondria. Enzyme deficiencies in the Krebs cycle have been rarely reported as the cause of metabolic disease presentations on the assumption that loss-of-function mutations affecting these key enzymes appear to be typically incompatible with life. To date, only recessively inherited variants in *SDH* (succinate dehydrogenase), *FH* (fumarate hydratase [MIM: 136850]), and *ACO2* (aconitase 2 [MIM: 100850]) have been associated with human disease. SDH comprises two structural subunits (SDHA and SDHB), and two additional structural components (SDHC and SDHD) and two assembly factors (SDHAF1 and SDHAF2) are required for the biogenesis of complex II (succinate-ubiquinone oxidoreductase) of the RC. Recessively inherited *SDHA* variants are mainly associated with Leigh syndrome (MIM: 256000), and mutations in *SDHB* (MIM: 185470), *SDHD* (MIM: 602690), and *SDHAF1* (MIM: 612848) are involved in severe infantile neurological presentations or cardiomyopathy associated with significant complex II deficiency.[9–12] Fumarase deficiency, associated with recessively inherited *FH* variants, is a metabolic disorder characterized by neurological impairment in early childhood accompanied by encephalopathy and seizures, often leading to death in the first years of life (for a review, see Ottolenghi et al.[13]). Finally, mutations in *ACO2*, encoding mitochondrial aconitase, are responsible for cerebellar-retinal degeneration in childhood.[14]

Our report documents that loss-of-function mutations in *MDH2* are compatible with life and are associated with early-onset severe neurological disease, and the deleterious nature of the identified *MDH2* variants has been confirmed by functional assessment in both human cells and a yeast model. Establishing the diagnosis of MDH2 deficiency is difficult. The clinical phenotypes and elevated lactate levels observed in all three children were highly evocative of an underlying mitochondrial disorder, although brain MRI findings were non-specific. However, we observed no sign of RC deficiency or mitochondrial dysfunction in fibroblasts from S1. Only cells from S3 showed a reduced activity of complex I, emphasizing the problems associated with RC analyses, including primary and secondary effects and tissue variation. As previously suggested,[15,16] functional splitting of the Krebs cycle in complementary mini-cycles might allow conversion of pyruvate to α-ketoglutarate even when there is a defect in the second half of the Krebs cycle. This would then produce reduced equivalents to sustain RC activity. In all affected individuals, levels of urinary malate and fumarate were moderately increased or normal (Figure S3). As observed in subject fibroblasts, we saw no increase in urinary succinate, except during an episode of metabolic decompensation in S2. It is important to bear in mind that MDH2-deficient individuals can show nearly normal concentrations of urinary organic acids and that analysis has to be repeated at the time of metabolic decompensation. There are no curative treatments for subjects with MDH2 deficiency, but trial of a ketogenic diet has appeared to decrease the frequency of epileptic seizures in two children, as has already been reported in other cases of refractory epilepsy, including that of a mitochondrial origin.[17,18]

Disruption of the Krebs cycle is also a hallmark of some cancers. Dominant defects associated with tumor development have been reported in three Krebs cycle enzymes: isocitrate dehydrogenase 1 (IDH1), SDH, and fumarate hydratase (FH),[19–21] and *MDH2* was also recently identified as a pheochromocytoma and paraganglioma susceptibility gene.[4] In subjects' paragangliomas, loss of heterozygosity was associated with loss of MDH2 activity, but surprisingly, this did not lead to malate accumulation. However, targeted knockdown of *MDH2* expression in HeLa cells by small hairpin RNA did lead to the accumulation of both malate and fumarate.[4] Like succinate and fumarate, malate stabilizes hypoxia inducible factor 1 alpha (HIF-1α), thus triggering the hypoxia response transcriptional program.[22] HIF-1α activation was found to induce transcriptional upregulation in some of the proteins participating in pyruvate metabolism, leading to PDH complex inactivation and pyruvate accumulation in the cytosol. Impairment of the PDH complex leads to a decrease in the production of NADH and FADH2, which donate electrons to the RC to complete the OXPHOS by generating ATP.[23] Thus, cells develop lactic acidosis as a result of a defective PDH complex, Krebs cycle, or RC. Secondary RC deficiency could also contribute to the mitochondrial phenotypes found in our subjects.

Although the recessive *MDH2* variants identified in the affected subjects described in this study lead to a severe neurological presentation, the fact that their parents are heterozygous carriers could place them at an elevated risk

of tumorigenesis. The parents of individuals presenting with *FH* recessive disorders often develop tumors.[24] For heterozygous carriers of mutations in the different *SDH* genes associated with infantile neurological presentations, the situation is unclear. Although there is no indication of cancer susceptibility in either family with *MDH2* mutations, we are recommending ongoing surveillance and screening.

In conclusion, we have shown that bi-allelic variants in *MDH2*, encoding a mitochondrially targeted Krebs cycle enzyme, are responsible for a severe infantile neurological phenotype. Although the clinical presentation and elevated lactate concentrations are highly evocative of mitochondrial disease, there are no specific biomarkers to pinpoint the rapid diagnosis of MDH2 deficiency, and as such, gene-panel or whole-exome sequencing analysis will not only be important for diagnosing further cases but also allow the detection of heterozygous carriers, thus determining their risk of tumorigenesis.

Accession Numbers

The data reported in this paper have been deposited in ClinVar (as three independent submissions corresponding to each subject or organization) under accession numbers ClinVar: SCV000321300 and SCV000321301for c.398C>T and c.620C>T, respectively; SCV000322707 and SCV000322708 for c.398C>T and c.596delG, respectively; and SCV000299301 for c.109G>A and c.398C>T.

Supplemental Data

Supplemental Data include three figures and three tables and can be found with this article online at http://dx.doi.org/10.1016/j.ajhg.2016.11.014.

Acknowledgments

We acknowledge Charlotte Cochaud and Pascal Colosetti for technical help, Hiromi Sesaki for providing the lentiviral vectors, and Mercedes Robledo for helpful discussion. We also thank the Pasteur-IRCAN Cellular and Molecular Imaging platform. This work was made possible by grants from the Fondation pour la Recherche Médicale and Fondation Maladies Rares to V.P.-F. This study was supported by the German Bundesministerium für Bildung und Forschung (BMBF) through the E-Rare project GENOMIT (01GM1603 to H.P. and A.R.). T.B.H. and J.G. were supported by the BMBF through the Juniorverbund in der Systemmedizin “mitOmics” (FKZ 01ZX1405C). H.P. was supported by the EU FP7 Mitochondrial European Educational Training Project (317433) and, together with J.G., by the EU Horizon2020 Collaborative Research Project SOUND (633974). R.W.T. was supported by a Wellcome Trust Strategic Award (096919/Z/11/Z), the Medical Research Council Centre for Neuromuscular Diseases (G0601943), the Lily Foundation, and the UK National Health Service Highly Specialised “Rare Mitochondrial Disorders of Adults and Children” Service in Newcastle upon Tyne. P.E.B. was supported by the NIH National Institute of Neurological Disorders and Stroke (R01NS083726).

Received: June 21, 2016
Accepted: November 16, 2016
Published: December 15, 2016

Web Resources

1000 Genomes, http://www.1000genomes.org/data
ClinVar, https://www.ncbi.nlm.nih.gov/clinvar/
dbSNP, http://www.ncbi.nlm.nih.gov/snp
ExAC Browser, http://exac.broadinstitute.org/
GenBank, http://www.ncbi.nlm.nih.gov/genbank/
GeneMatcher, http://genematcher.org
HapMap, https://hapmap.ncbi.nlm.nih.gov/
MitoCarta, http://www.broadinstitute.org/files/shared/metabolism/mitocarta/human.mitocarta2.0.html
MutationTaster, http://www.mutationtaster.org
NHLBI Exome Sequencing Project (ESP) Exome Variant Server, http://evs.gs.washington.edu/EVS/
OMIM, http://www.omim.org/
PolyPhen-2, http://genetics.bwh.harvard.edu/pph2/
RefSeq, http://www.ncbi.nlm.nih.gov/refseq/
SIFT, http://sift.jcvi.org/
Swiss-Pdb Viewer 3.7, http://www.expasy.org/pdb

References

1. Khurana, D.S., Salganicoff, L., Melvin, J.J., Hobdell, E.F., Valencia, I., Hardison, H.H., Marks, H.G., Grover, W.D., and Legido, A. (2008). Epilepsy and respiratory chain defects in children with mitochondrial encephalopathies. Neuropediatrics *39*, 8–13.
2. Sobreira, N., Schiettecatte, F., Valle, D., and Hamosh, A. (2015). GeneMatcher: a matching tool for connecting investigators with an interest in the same gene. Hum. Mutat. *36*, 928–930.
3. Bannwarth, S., Ait-El-Mkadem, S., Chaussenot, A., Genin, E.C., Lacas-Gervais, S., Fragaki, K., Berg-Alonso, L., Kageyama, Y., Serre, V., Moore, D.G., et al. (2014). A mitochondrial origin for frontotemporal dementia and amyotrophic lateral sclerosis through CHCHD10 involvement. Brain *137*, 2329–2345.
4. Cascón, A., Comino-Méndez, I., Currás-Freixes, M., de Cubas, A.A., Contreras, L., Richter, S., Peitzsch, M., Mancikova, V., Inglada-Pérez, L., Pérez-Barrios, A., et al. (2015). Whole-exome sequencing identifies MDH2 as a new familial paraganglioma gene. J. Natl. Cancer Inst. *107*. http://dx.doi.org/10.1093/jnci/djv053.
5. Lasserre, J.-P., Dautant, A., Aiyar, R.S., Kucharczyk, R., Glatigny, A., Tribouillard-Tanvier, D., Rytka, J., Blondel, M., Skoczen, N., Reynier, P., et al. (2015). Yeast as a system for modeling mitochondrial disease mechanisms and discovering therapies. Dis. Model. Mech. *8*, 509–526.
6. Westermann, B., and Neupert, W. (2000). Mitochondria-targeted green fluorescent proteins: convenient tools for the study of organelle biogenesis in Saccharomyces cerevisiae. Yeast *16*, 1421–1427.
7. Rouzier, C., Bannwarth, S., Chaussenot, A., Chevrollier, A., Verschueren, A., Bonello-Palot, N., Fragaki, K., Cano, A., Pouget, J., Pellissier, J.-F., et al. (2012). The MFN2 gene is responsible for mitochondrial DNA instability and optic atrophy ‘plus’ phenotype. Brain *135*, 23–34.
8. Robinson, B.H., Petrova-Benedict, R., Buncic, J.R., and Wallace, D.C. (1992). Nonviability of cells with oxidative

defects in galactose medium: a screening test for affected patient fibroblasts. Biochem. Med. Metab. Biol. *48*, 122–126.
9. Bourgeron, T., Rustin, P., Chretien, D., Birch-Machin, M., Bourgeois, M., Viegas-Péquignot, E., Munnich, A., and Rötig, A. (1995). Mutation of a nuclear succinate dehydrogenase gene results in mitochondrial respiratory chain deficiency. Nat. Genet. *11*, 144–149.
10. Alston, C.L., Davison, J.E., Meloni, F., van der Westhuizen, F.H., He, L., Hornig-Do, H.-T., Peet, A.C., Gissen, P., Goffrini, P., Ferrero, I., et al. (2012). Recessive germline SDHA and SDHB mutations causing leukodystrophy and isolated mitochondrial complex II deficiency. J. Med. Genet. *49*, 569–577.
11. Jackson, C.B., Nuoffer, J.-M., Hahn, D., Prokisch, H., Haberberger, B., Gautschi, M., Häberli, A., Gallati, S., and Schaller, A. (2014). Mutations in SDHD lead to autosomal recessive encephalomyopathy and isolated mitochondrial complex II deficiency. J. Med. Genet. *51*, 170–175.
12. Ghezzi, D., Goffrini, P., Uziel, G., Horvath, R., Klopstock, T., Lochmüller, H., D'Adamo, P., Gasparini, P., Strom, T.M., Prokisch, H., et al. (2009). SDHAF1, encoding a LYR complex-II specific assembly factor, is mutated in SDH-defective infantile leukoencephalopathy. Nat. Genet. *41*, 654–656.
13. Ottolenghi, C., Hubert, L., Allanore, Y., Brassier, A., Altuzarra, C., Mellot-Draznieks, C., Bekri, S., Goldenberg, A., Veyrieres, S., Boddaert, N., et al. (2011). Clinical and biochemical heterogeneity associated with fumarase deficiency. Hum. Mutat. *32*, 1046–1052.
14. Spiegel, R., Pines, O., Ta-Shma, A., Burak, E., Shaag, A., Halvardson, J., Edvardson, S., Mahajna, M., Zenvirt, S., Saada, A., et al. (2012). Infantile cerebellar-retinal degeneration associated with a mutation in mitochondrial aconitase, ACO2. Am. J. Hum. Genet. *90*, 518–523.
15. Yudkoff, M., Nelson, D., Daikhin, Y., and Erecińska, M. (1994). Tricarboxylic acid cycle in rat brain synaptosomes. Fluxes and interactions with aspartate aminotransferase and malate/aspartate shuttle. J. Biol. Chem. *269*, 27414–27420.
16. Rustin, P., Bourgeron, T., Parfait, B., Chretien, D., Munnich, A., and Rötig, A. (1997). Inborn errors of the Krebs cycle: a group of unusual mitochondrial diseases in human. Biochim. Biophys. Acta *1361*, 185–197.
17. Pasca, L., De Giorgis, V., Macasaet, J.A., Trentani, C., Tagliabue, A., and Veggiotti, P. (2016). The changing face of dietary therapy for epilepsy. Eur. J. Pediatr. *175*, 1267–1276.
18. Kang, H.-C., Lee, Y.-M., Kim, H.D., Lee, J.S., and Slama, A. (2007). Safe and effective use of the ketogenic diet in children with epilepsy and mitochondrial respiratory chain complex defects. Epilepsia *48*, 82–88.
19. Baysal, B.E., Ferrell, R.E., Willett-Brozick, J.E., Lawrence, E.C., Myssiorek, D., Bosch, A., van der Mey, A., Taschner, P.E., Rubinstein, W.S., Myers, E.N., et al. (2000). Mutations in SDHD, a mitochondrial complex II gene, in hereditary paraganglioma. Science *287*, 848–851.
20. Tomlinson, I.P.M., Alam, N.A., Rowan, A.J., Barclay, E., Jaeger, E.E.M., Kelsell, D., Leigh, I., Gorman, P., Lamlum, H., Rahman, S., et al.; Multiple Leiomyoma Consortium (2002). Germline mutations in FH predispose to dominantly inherited uterine fibroids, skin leiomyomata and papillary renal cell cancer. Nat. Genet. *30*, 406–410.
21. Yan, H., Parsons, D.W., Jin, G., McLendon, R., Rasheed, B.A., Yuan, W., Kos, I., Batinic-Haberle, I., Jones, S., Riggins, G.J., et al. (2009). IDH1 and IDH2 mutations in gliomas. N. Engl. J. Med. *360*, 765–773.
22. Raimundo, N., Baysal, B.E., and Shadel, G.S. (2011). Revisiting the TCA cycle: signaling to tumor formation. Trends Mol. Med. *17*, 641–649.
23. Jochmanová, I., Zhuang, Z., and Pacak, K. (2015). Pheochromocytoma: Gasping for Air. Horm. Cancer *6*, 191–205.
24. Bayley, J.-P., Launonen, V., and Tomlinson, I.P.M. (2008). The FH mutation database: an online database of fumarate hydratase mutations involved in the MCUL (HLRCC) tumor syndrome and congenital fumarase deficiency. BMC Med. Genet. *9*, 20.

The American Journal of Human Genetics, Volume *100*

Supplemental Data

Mutations in *MDH2*, Encoding a Krebs Cycle Enzyme, Cause Early-Onset Severe Encephalopathy

Samira Ait-El-Mkadem, Manal Dayem-Quere, Mirjana Gusic, Annabelle Chaussenot, Sylvie Bannwarth, Bérengère François, Emmanuelle C. Genin, Konstantina Fragaki, Catharina L.M. Volker-Touw, Christelle Vasnier, Valérie Serre, Koen L.I. van Gassen, Françoise Lespinasse, Susan Richter, Graeme Eisenhofer, Cécile Rouzier, Fanny Mochel, Anne De Saint-Martin, Marie-Thérèse Abi Warde, Monique G.M. de Sain-van der Velde, Judith J.M. Jans, Jeanne Amiel, Ziga Avsec, Christian Mertes, Tobias B. Haack, Tim Strom, Thomas Meitinger, Penelope E. Bonnen, Robert W. Taylor, Julien Gagneur, Peter M. van Hasselt, Agnès Rötig, Agnès Delahodde, Holger Prokisch, Sabine A. Fuchs, and Véronique Paquis-Flucklinger

SUPPLEMENTAL FIGURES

Figure S1.

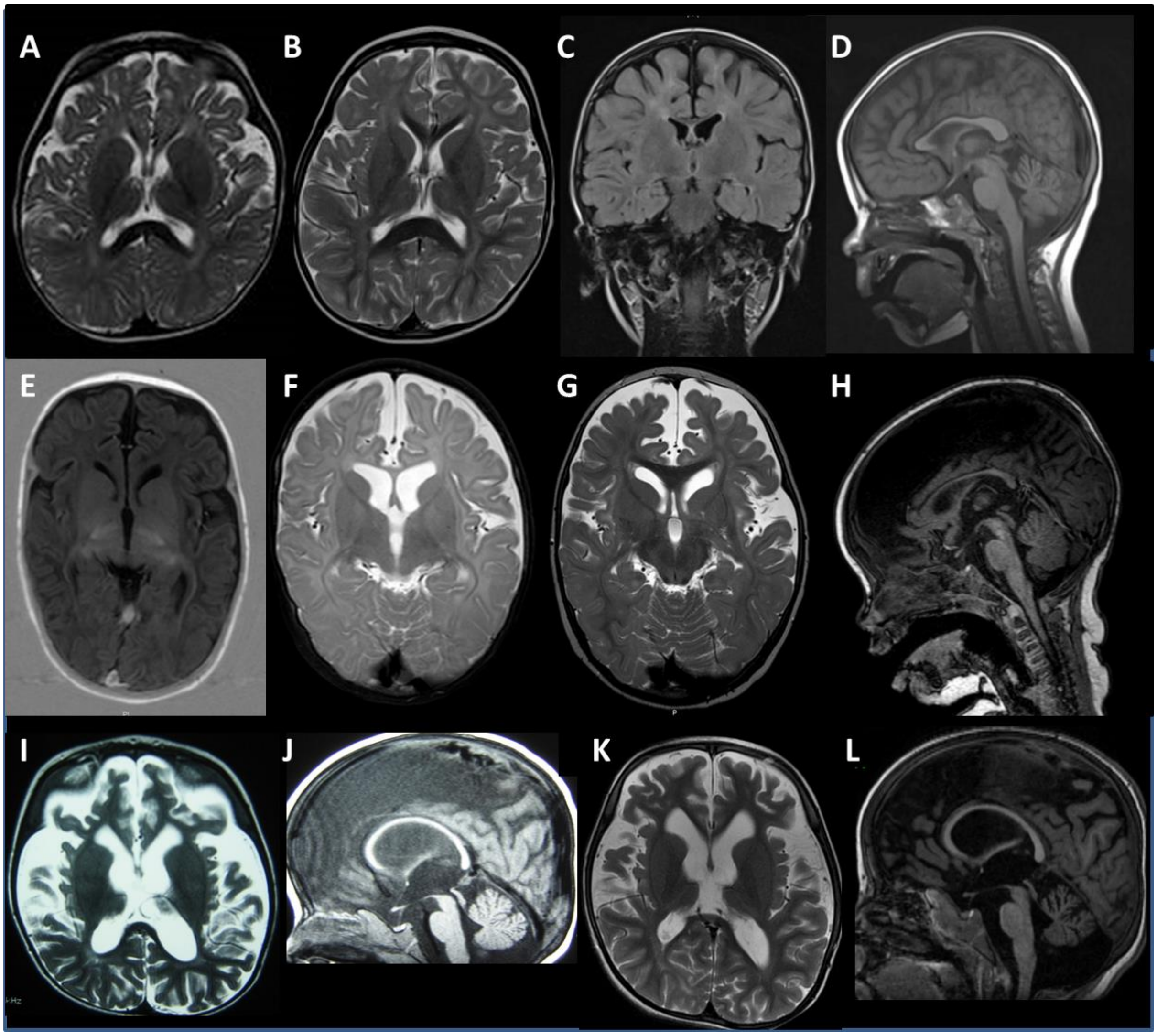

Brain MRI of Subject 1 (A-D), Subject 2 (E-H) and Subject 3 (I-L). Subject 1: Supratentorial cortical atrophy mainly in the frontal and parietal lobes at one year of age (A) and at 2.5 years of age (B-C) with thinning of the anterior part of the corpus callosum (D). Subject 2: normal MRI at 3 months of age (E). Gradual cortical and subcortical atrophy in the frontal lobes at 6 months of age (F) with parietal involvement at one year of age (G) and thinning of the genu of the corpus callosum (H). Subject 3: Cerebral cortical and subcortical atrophy at 18 months of age (I-J) associated with cerebellar atrophy at 3.5 years (K-L).

Figure S2.

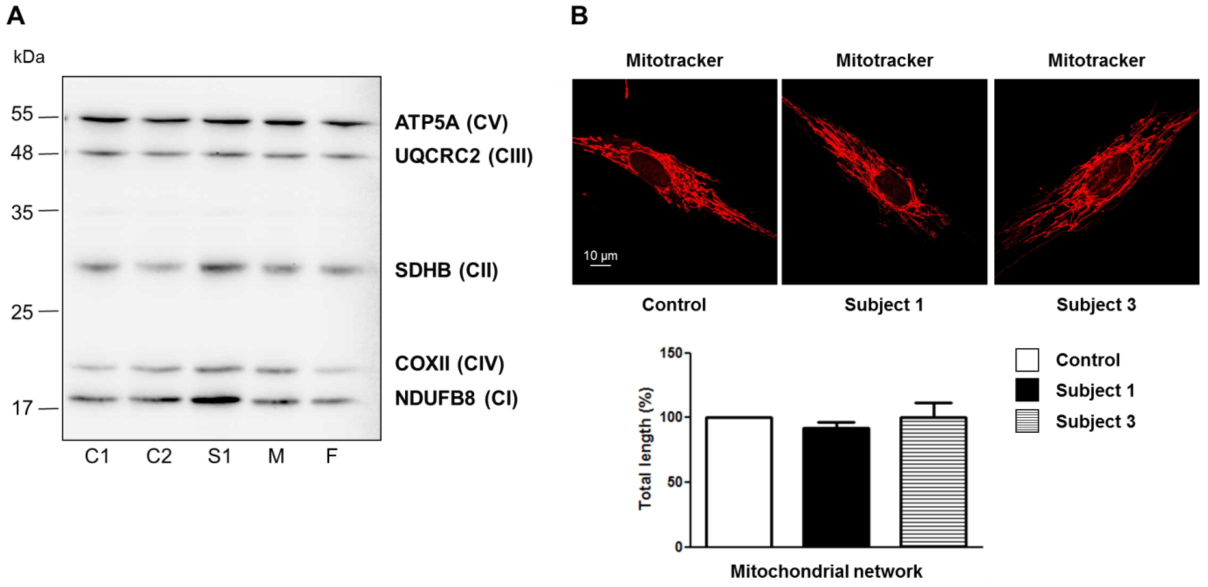

OXPHOS levels and mitochondrial network in fibroblasts from affected children. A. Representative western blot of ATP5A, UQCRC2, SDHB, COX II, and NDUFB8 performed with fibroblast lysates obtained from two control individuals (C1, C2), the affected subject (S1), his mother (M) and his father (F). A cocktail of mouse anti-human total OXPHOS complex antibodies (Abcam, ab110411) was used at 1/1000 for the blot. CI, complex I; CII, complex II ; CIII, complex III ; CVI, complex VI ; CV, complex V. **B.** Cells obtained from a control and the affected subjects (S1 and S3) were analyzed by confocal microscopy using Mitotracker Red (upper panel) as previously described.[1] Mitochondrial phenotypes were quantified for 35 randomly-selected individual cells per each studied fibroblast cell line from 3 independent experiments (lower panel). The data obtained were used to calculate the total length of the mitochondrial network per cell. Differences between the cell lines were analyzed by Student’s t-test: ns, non significant.

Figure S3.

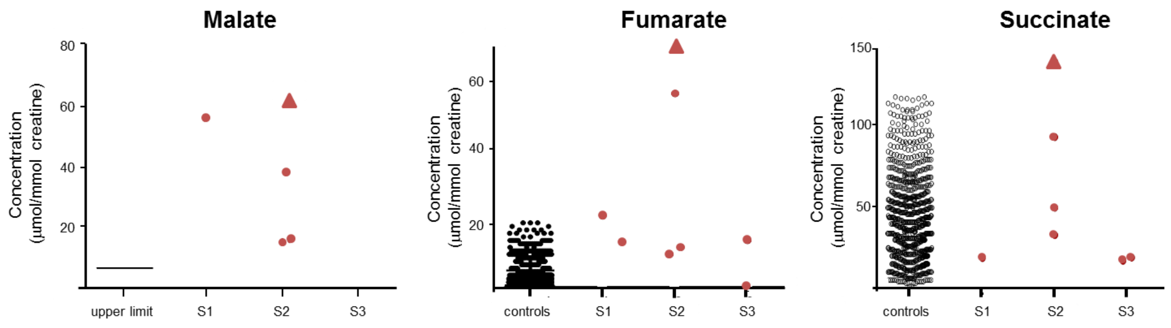

Urinary malate, fumarate and succinate excretion in the affected subjects. S1-3, Subjects 1-3; reference values (malate: from literature, fumarate n=728 and succinate n>1000) representing the 95% confidence interval of all analyses performed in Utrecht metabolic laboratory in boys (age 0-4 years) from January 2010 to May 2016. Circle, in normal conditions; triangle, during metabolic decompensation.

SUPPLEMENTAL TABLES

Table S1. Respiratory chain analysis in samples from Subjects 1 and 3. Spectrophotometric analysis on muscle (A), liver (B) of Subject 1 and muscle of Subject 3 (C). Spectrophotometric analysis on fibroblasts of Subjects 1 and 3 cultivated in glucose (D) and galactose (F) medium. Polarographic analysis of the respiratory chain enzyme activities in fibroblasts of Subjects 1 and 3 cultivated in glucose (E) and in galactose medium (G). Results are expressed as extreme absolute values or absolute values for controls or affected subjects, respectively. Lower values are highlighted in grey. nd, not done. G3P = glycerol 3-phosphate. CS = citrate synthase. LDH = lactate dehydrogenase. Spectrophotometric measurements and polarographic studies were performed as previously described.[2]

A

SPECTROPHOTOMETRIC ANALYSIS ON MUSCLE							
Enzymatic activities	I	II	III	IV	V	CS	LDH
Control values (nmol/min/mg of proteins)	10-32	20-65	94-236	89-347	32-89	82-234	1259-7234
Subject 1	22.5	36.9	232.9	255.7	50.3	175.6	4106

B

SPECTROPHOTOMETRIC ANALYSIS ON LIVER							
Enzymatic activities	I	II	III	IV	V	CS	LDH
Control values (nmol/min/mg of proteins)	13-41	78-267	124-246	146-316	41-117	43-107	736-2359
Subject 1	35.5	157.0	154.0	246.5	39.7	77.8	1438

C

SPECTROPHOTOMETRIC ANALYSIS ON MUSCLE							
Enzymatic activities	I	II	III	IV	V	CS	LDH
Control values (nmol/min/mg of proteins)	40-95	66-153	925-2068	418-1210	165-414	82-234	1259-7234
Subject 3	68.0	nd	1820.0	920.0	122.0	nd	nd

D

SPECTROPHOTOMETRIC ANALYSIS ON FIBROBLASTS GROWN IN GLUCOSE MEDIUM							
Enzymatic activities	I	II	III	IV	V	CS	LDH
Control values (nmol/min/mg of proteins)	9.0-27.1	18.5-54.0	57.4-176.2	109.9-350.0	22.0-46.2	74.7-161.1	2642-6971
Subject 1	13.3	26.8	100.3	229.9	45.0	137.3	4022
Control values (nmol/min/mg of proteins)	27-44	56-83	547-846	285-436	53-84	74.7-161.1	2642-6971
Subject 3	27.0	145.0	1140.0	678.0	95.0	188.6	4601

E

POLAROGRAPHIC ANALYSIS ON FIBROBLASTS GROWN IN GLUCOSE MEDIUM				
Oxygen consumption	Intact cells	Digitonin permeabilized cells		
		Glutamate+Malate	Succinate	G3P
Control values (nmol O2/min/mg of proteins)	5.90-13.80	8.00-16.60	8.00-15.80	4.90-13.50
Subject 1	10.40	8.82	8.66	9.70
Subject 3	9.85	12.52	10.75	9.95

F

SPECTROPHOTOMETRIC ANALYSIS ON FIBROBLASTS GROWN IN GALACTOSE MEDIUM							
Enzymatic activities	I	II	III	IV	V	CS	LDH
Control values (nmol/min/mg of proteins)	15.2-20.1	28.2-48.0	88.8-143.0	181.7-315.4	22.7-47.5	124.8-225.0	3100-6100
Subject 1	18.3	29.8	114.1	253.4	32.5	134.4	5816
Subject 3	23.4	62.6	224.8	362.8	48.6	238.0	4019

G

POLAROGRAPHIC ANALYSIS ON FIBROBLASTS GROWN IN GALACTOSE MEDIUM				
Oxygen consumption	Intact cells	Digitonin permeabilized cells		
		Glutamate+Malate	Succinate	G3P
Control values (nmol O2/min/mg of proteins)	5.58-17.44	8.16-13.45	8.60-10.97	5.22-12.91
Subject 1	15.51	11.00	10.03	9.49
Subject 3	29.27	18.83	16.87	17.65

Table S1

Table S2. Bioinformatics analysis of *MDH2* missense variants.

* : UCSC GRCh37/hg19 assembly

D: damaging or disease causing

CADD: Combined Annotation Dependent Depletion

GERP: Genomic Evolutionary Rate Profiling

PhyloP: phylogenetic p-values

# Chr	Genomic position*	NM-ID	cDNA change	Protein change	SIFT score	SIFT prediction	Polyphen2 _hdiv score	Polyphen2 _hdiv prediction	MutationTaster score	MutationTaster prediction	CAAD_Phred	GERP_RS	PhyloP score
7	75684190	NM_005918	G109A	G37R	0,99	D	1	D	1	D	34	5,83	2,76
7	75687365	NM_005918	C398T	P133L	1	D	1	D	1	D	18,3	5,56	2,9
7	75692897	NM_005918	C620T	P207L	1	D	1	D	1	D	21,8	5,69	2,69

Table S3. Sequences of primers used for Sanger sequencing and complementation studies

Name	Sequence
Primers used for Sanger sequencing of MDH2 variants	
MDH2-Ex2F	gaggcccagtgcaacattat
MDH2-Ex2R	gaaacccccgtctctaaaca
MDH2-Ex4F	atttcctgtaacagctggcg
MDH2-Ex4R	gcacatcagcagcctttg
MDH2-Ex6F	ggcagctgtgacatgttttac
MDH2-Ex6R	Aggccttctccagtctacc
Primers used for molecular cloning of human MDH2 cDNA	
MDH2-BclI-F	ttccatgatcacccgctccagccatgct
MDH2-NotI-R	actttgcggccgctcacttcagggtctt
Primers used for Sanger sequencing of human MDH2 cDNA	
MDH2-cDNA-F1	gacgacctgttcaacaccaa
MDH2-cDNA-R1	gtgttggctctgacgatgtc
MDH2-cDNA-F2	tggatccagctcgagtcaa
MDH2-cDNA-R2	gcggtgtggagaagtaggta
MDH2-cDNA-F3	aagggcatcgagaagaacct
MDH2-cDNA-R3	aggcggctcaccaaggggc
Primers used for yeast studies	
F1-MDH1	ctttaaaggacacagacatggttttaattccggatccccgggttaattaa
R1-MDH1	tttcgatattcttcttcaaggtttctttacgaattcgagctcgtttaaac
MDH2-Eco	cggatttccgatgctctccgccctcgtcc
MDH2-Hind	cccaagcttgggtcacttcagggtcttcacg
MDH1-Recfw	ctataactacaaaaaacacatacaggaattagcttacgcgtcgagcccggaattccgatgttgtcaagagtagctaaac
MDH1-RecRev	aaggaaaaacgttcattgttccttattcagttagctagctcccaagcttgggtcacttcctatttactagcaacaaagttg
mdh1-P128Lfw	accgccgaatccgctctcaatgctgccattctg
mdh1-P128LRev	cagaatggcagcattgagagcggattcggcggt
mdh1-P202Lfw	ggtattaccatcatcctattgatttcgcaaaca
mdh1-P202LRev	tgtttgcgaaatcaataggatgatggtaatacc
mdh1-G30Rfw	gcaggcggtggtattggacaaccattg
mdh1-PG30RRev	caatggttgtccaataccaccgcctgc

SUPPLEMENTAL REFERENCES

1. Bannwarth, S., Ait-El-Mkadem, S., Chaussenot, A., Genin, E.C., Lacas-Gervais, S., Fragaki, K., Berg-Alonso, L., Kageyama, Y., Serre, V., Moore, D.G., et al. (2014). A mitochondrial origin for frontotemporal dementia and amyotrophic lateral sclerosis through CHCHD10 involvement. Brain *137*, 2329–2345.

2. Rustin, P., Chretien, D., Bourgeron, T., Gérard, B., Rötig, A., Saudubray, J.M., and Munnich, A. (1994). Biochemical and molecular investigations in respiratory chain deficiencies. Clin. Chim. Acta Int. J. Clin. Chem. *228*, 35–51.
